# Effects of the Ketogenic Diet on Strength Performance in Trained Men and Women: A Systematic Review and Meta-Analysis

**DOI:** 10.3390/nu16142200

**Published:** 2024-07-10

**Authors:** Salvador Vargas-Molina, Mora Murri, Andrés Gonzalez-Jimenez, José Luis Gómez-Urquiza, Javier Benítez-Porres

**Affiliations:** 1 Physical Education and Sports Area, Faculty of Medicine, University of Málaga, 29010 Málaga, Spain; salvadorvargasmolina@gmail.com; 2 Research Division, Dynamical Business and Science Society—DBSS International SAS, Bogotá 110311, Colombia; 3 Instituto de Investigacion Biomedica de Malaga, IBIMA-Plataforma BIONAND, 29590 Málaga, Spain; bioinformatica@ibima.eu; 4 Endocrinology and Nutrition Clinical Management Unit, Virgen de la Victoria University Hospital, 29010 Málaga, Spain; 5 Centro de Investigación Biomédica en Red de la Fisiopatología de la Obesidad y la Nutrición (CIBERObn), Instituto de Salud Carlos III, 29016 Madrid, Spain; 6 Department of Nursing, Faculty of Health Sciences, University of Granada, 51005 Ceuta, Spain; jlgurquiza@ugr.es

**Keywords:** carbohydrate restriction, ketosis, performance, repetition maximum, resistance-training, strength parameters

## Abstract

Ketogenic diets (KDs) are an alternative to improve strength performance and body composition in resistance training participants. The objective of this review and meta-analysis is to verify whether a ketogenic diet produces an increase in the strength of resistance-trained participants. We have evaluated the effect of the ketogenic diet in conjunction with resistance training on the strength levels in trained participants. Boolean algorithms from various databases (PubMed, Scopus, and Web of Science) were used. Meta-analyses were carried out, one on the 1-RM squat (SQ), with 106 trained participants or athletes, and another on the 1-RM on the bench press (BP), evaluating 119 participants. We did not find significant differences between the groups in the variables of SQ or BP, although the size of the effect was slightly higher in the ketogenic group. Conclusions: KDs do not appear to impair 1-RM performance; however, this test does not appear to be the most optimal tool for assessing hypertrophy-based strength session performance in resistance-trained participants.

## 1. Introduction

Ketogenic diets (KDs), based on a reduction in carbohydrates (CHO) equivalent to 5–10% of total caloric intake or below 20–50 g/day while fat is increased [1,2], have been proposed as an alternative for increasing muscle mass in recreational or advanced participants engaged in resistance training (RT). However, we should keep in mind that the main recommendations to increase strength and muscle hypertrophy recommend 4–7 g/kg/day of body weight of carbohydrates [3], or even 8–10 g/kg/day of bodyweight when anaerobic work is performed [4], as is the case with strength training.

**Figure 1:**
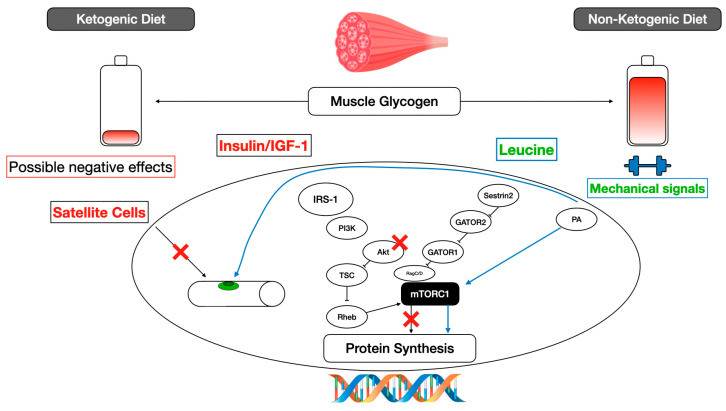
Application of ketogenic diet and resistance training. Adapted from [6]. IGF-1 (insulin-like growth factor); IRS-1 (insulin receptor substrate-1); Akt (protein kinase B); TSC (tuberous sclerosis protein); GATOR (GAP activity toward RAGs); mTOR (mechanistic target of rapamycin); RHEB (Ras homolog enriched in brain); PI3K (phosphoinositide 3-kinase); PA (phosphatidic acid).

In our previous work [5], we reported that KD can be an alternative for increasing fat free mass (FFM) in advanced participants trained in RT, as energy surplus is generated. However, it does not seem to be the best option for muscle hypertrophy, due to the effect of satiety and the lack of adherence that is generated. Figure 1 shows how strength exercise can activate protein signaling pathways through mTORC1 or satellite cells. However, when a KD is applied, the signaling chains can be inhibited in Akt or in the activation of satellite cells themselves. Furthermore, insulin or insulin growth factor does not seem to optimize its function under a ketogenic condition [6]. On the other hand, KDs generate a very high concentration of beta-hydroxybutyrate, and it has been reported that this metabolite can reduce leucine oxidation, thereby favoring the preservation of muscle mass [7]. Interestingly, beta-hydroxybutyrate has been shown to have neuroprotective effects, specifically in BV2 cells, inducing microglial polarization, significantly reducing the expression levels of the pro-inflammatory cytokine IL-17 and increasing the levels of the anti-inflammatory cytokine IL-10 [8].

In order to increase muscle hypertrophy, a manipulation of programming variables is required to optimize the performance of trained participants in the RT sessions [9,10]. We should consider mechanical tension as the main physiological mechanism or even as the only one that favors muscle hypertrophy [11]. By mechanical tension we mean a minimum mechanical load that triggers the synthesis of myofibrillar proteins, stipulated at a minimum threshold greater than 30% of the maximum repetition [11] and with the volume as the most determining programming variable [12], understanding volume as the number of total sets performed in the session or week [13]. Additionally, we should keep in mind that the repetition range of 6 to 12 is not the only range that can generate muscle tissue increase [10]. Therefore, a plausible reasoning would be that a KD should affect neither the volume nor the intensity. However, dietary carbohydrates can improve performance in endurance sports [14]. In long-term sports, in some cases, it is intended that fat be used as fuel, as it contains an energy reserve higher than that of carbohydrates [15]. For this reason, carbohydrate-restricted diets are used, as the muscular system stores less glycogen, and the use of fat as fuel is promoted [16]. Nevertheless, a traditional RT based on hypertrophy requires a higher volume (more series and more repetitions) [9] and so a reduction in carbohydrates can impair performance. However, it has been shown that a training session with no more than 10 total sets does not seem to affect performance [17]. The average number of sets per muscle group in a training session aimed at hypertrophy is speculated to be 10 sets, with a total of 20 sets per week [18], distributed over a frequency of two days per week [19].

Recently, other parameters have been evaluated related to sports performance during a strength training session in advanced participants, applying 15 total series per session, although from different muscle groups, and variables such as volume load, number of total repetitions, weight loss, speed, and perception of effort [20], not finding any decrease in these variables over six weeks.

In addition, in order to know whether the KDs are optimally performed in advanced participants in resistance training, tests that have a direct transfer in the training session should be used. The one-repetition maximum test (1-RM) [21,22] has mainly been used for this purpose and may be optimal for modalities such as powerlifting or weightlifting [23] since the main energy consumption is phosphocreatine. This test has also been used for CrossFit^®^ (Santa Cruz, CA, USA) [24,25]. It is an evaluation parameter for which a minimum threshold of lifted load is established to assess whether a subject is advanced or not.

Among the limitations of evaluating performance through 1-RM is that the study subject must perform at maximum intention/effort; it will depend on the level of motivation he or she has at that moment [26]. In addition, heart rate or concentration can have a notable influence. Due to the inconsistency of this test in evaluating strength, it has been suggested that older adults should have between eight and nine test sessions [27].

However, even if 1-RM has been used for evaluating the strength in all the included studies of the present meta-analysis, it may not be the best option to identify the effect of a KD on the performance of participants who engage in RT with a goal of muscle hypertrophy.

In fact, other investigations in addition to 1-RM have incorporated different variables that can further define the objective of this study population, such as the number of repetitions [28] or the load volume [22].

Our present review and meta-analysis has a double objective: (a) to evaluate the effect of ketosis on strength levels, and (b) to verify whether the performance parameters applied for this type of population, participants who train using RT, are optimal.

## 2. Materials and Methods

The present review and meta-analysis was conducted according to PRISMA [29]. The protocol was registered in PROSPERO (CRD42023422743). Additionally, the PEDro scale [30] was employed in order to quickly assess whether the studies present reliable and meaningful results

### 2.1. Search Strategy

A systematic review of the literature was conducted using online medical databases, including Web of Science, PubMed/Medline, and SCOPUS. Studies published up to April 2022 were identified and analyzed. The following search terms were used: (“Ketogenic diet” OR “ketogenic dieting” OR “low-carbohydrate diet” OR “low carbohydrate ketogenic diet” OR “very-low-carbohydrate diet” OR “cyclical ketogenic”) AND (“repetition maximum” OR “performance” OR “countermovement jump” OR “squat jump” OR “Squat” OR “bench press” OR “RM” OR “CMJ” OR “SJ”).

### 2.2. Study Selection

The inclusion criteria included: (a) the use of a KD in participants, competitors or elite strength-trained athletes; (b) randomized trials, with a minimum duration of eight weeks; (c) force evaluation; (d) data presented as means and standard deviations; (e) no intervention with nutritional or dietary supplements; and (f) articles written in English and available in their entirety. Exclusion criteria included: (a) research conducted on animals; (b) systematic reviews or meta-analyses or uncontrolled experimental studies; (c) research without control group; and (d) studies reporting fewer than x cases and/or controls. Study selection was performed independently by two investigators (S.V.M and J.B.P). Disagreements were discussed and resolved by consensus with a third investigator (M.M).

### 2.3. Data Extraction

The following data were extracted from the retrieved studies: publication date, country, authors’ names, experimental population, average age, gender, study design, duration of the protocols, composition of the diet in calories and macronutrients, means and standard deviations (SD). Data related to basic anthropometry and the evaluated strength were extracted.

### 2.4. Quality Assessment

The risk of bias assessment was evaluated using the Cochrane method [31]. The following were evaluated: random sequence generation, allocation concealment, blinding of participants and personnel, blinding of outcome assessment, incomplete outcome data, selective reporting, other bias. Studies were classified as high risk of bias, low risk of bias, or unclear bias for each item assessed, based on the recommendations of the Cochrane Handbook.

### 2.5. Metaanlysis

Two fixed-effect meta-analyses were conducted to examine the effect size (mean difference) in the 1-RM SQ and BP. Initially, the mean difference (pre- and post-exercise) and combined standard deviations, or error propagation, were calculated in both studies. Subsequently, the meta-analyses of continuous outcomes were carried out using the ‘metacon’ function from the ‘meta’ package in the R language (version 4.1.3). Heterogeneity was assessed using the *I*^2^ index, and publication bias was examined through Egger’s test and funnel plot analysis.

## 3. Results

**Figure 2:**
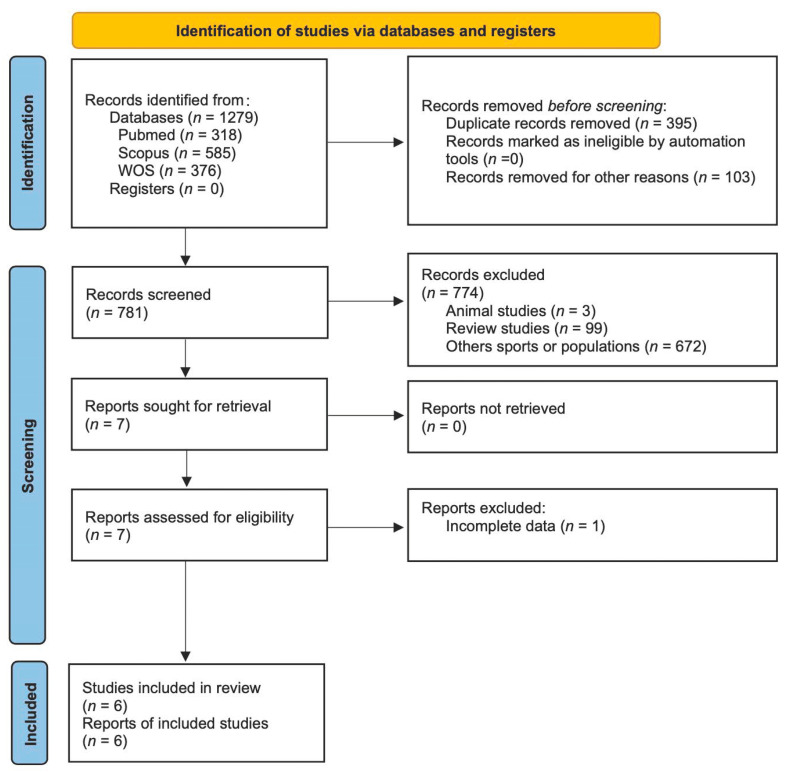
CONSORT Diagram.

Figure 2 shows the flowchart of our meta-analysis. We started with 1279 possible articles. Articles were checked and eligibility determined. We excluded 774 studies and included 6 studies in the final analysis. These studies included 67 participants on the KD and 64 participants in the control group.

The characteristics of these six controlled trials are summarized in Table 1. The study by Kysel et al. [32] was not included in the variable SQ and the article of Kephart et al. [33] was not included in the BP variable. The remaining four studies evaluated both BP and SQ. All the studies reported nutritional control and were carried out for a period between 8 and 12 weeks. It should be noted that the study of Greene et al. [23] was not included in our meta-analysis despite the fact that 1-RM was evaluated in SQ and BP, as both data were reported together.

**Figure 3:**
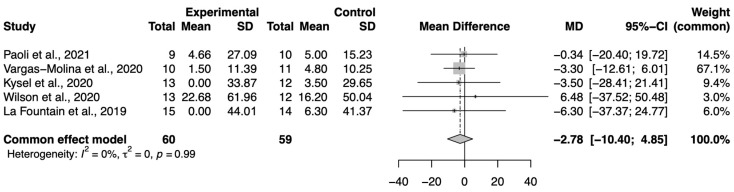
Forest plot of the bench press. SD: standard deviation; MD: mean difference; CI: confidence interval [21,22,28,32,34].

Five studies were included in the BP meta-analysis, with n = 60 in the experimental group and n = 59 in the control group. The effect size, mean difference, in the 1-RM was −2.78 (95%CI −10.40, 4.85) in favor of the control group; however, there were no significant differences (*p* > 0.05), as indicated in Figure 3.

**Figure 4:**
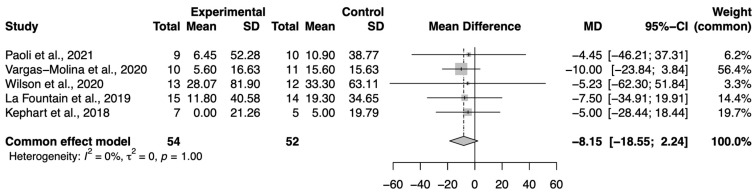
Forest plot of the squat. SD: standard deviation; MD: mean difference; CI: confidence interval [21,22,28,33,34].

Regarding the meta-analysis of 1-RM in SQ, the effect size was in favor of the control group (n = 54), compared to the control group (n = 52) of −8.15 (95%CI −18.55, 2.24) with *p* > of 0.05 (Figure 4).

The *I*^2^ value was 0% in both meta-analyses, and Egger’s test showed the absence of publication bias in both. Sensitivity analysis showed no changes in statistical significance or sense of effect size in any of the meta-analyses when one study was removed from the other results.

## 4. Discussion

Our present meta-analysis did not show significant differences in the KD group compared to the control group in the 1-RM in SQ and in BP; however, the effect size was slightly higher in the control group in both variables. A previous systematic review performed by Kang et al. [35] evaluated seven studies on the effect of KDs on strength-power. Three studies did not show significant effects on strength and power. Two studies showed an improvement in grip strength, abdominals, and 1-RM for SQ and BP, although without significant changes from the control group. Similar results were observed in the study of Murphy et al. [36], where 16 studies that related power or strength performance were selected. Three studies reported lower results, eleven studies found no difference between groups, and two studies found more optimal results in the KD group; however, the study population was not composed of advanced participants in resistance training.

**Figure 5:**
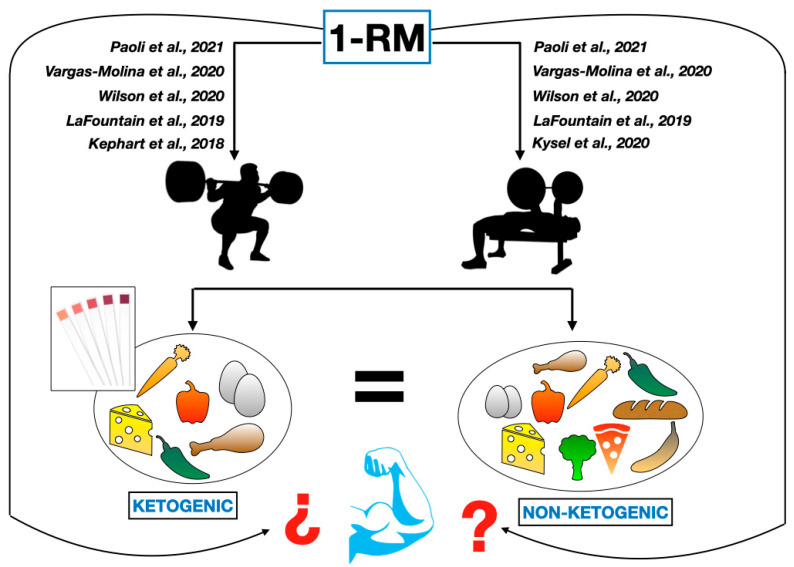
Ketogenic and non-ketogenic protocols with similar 1-RM results [21,22,28,33,34].

The only review and meta-analysis performed on trained participants, by Koerich et al. [37], showed more favorable effects for the carbohydrate-rich group than the KD on the 1-RM. However, this meta-analysis included studies on not only resistance training but also other sports modalities such as racewalking, endurance training, triathlons, cycling, and running. Therefore, it cannot be compared with a study of resistance-trained participants or strength athletes with a muscle hypertrophy profile. To summarize, Figure 5 shows the selected studies in each variable (1-RM BP and SQ).

However, the studies that have evaluated both body composition and strength performance have been based on the 1-RM test, where decreases in muscle performance have been found when expressed in absolute units and not in relation to the strength by total body mass (e.g., one repetition maximum) [38]. These types of tests are maximum and of short duration, so the substrate used would be the phosphagen [39], by which they can be useful in modalities such as powerlifting or weightlifting since the effort is equivalent; however, this is not the best option for participants who train RT and restrict carbohydrates, since performance could not be appreciated in a traditional session. In fact, there are variables that may be more optimal to assess performance in a session with the goal of hypertrophy in this population trained in RT. In this regard, Kephart et al. [33] evaluated the number of push-up repetitions and no significant differences between KD and control groups were found, although both improved. LaFountain et al. [28] evaluated the volume of performed repetitions and the total tonnage lifted in the exercises squat, deadlift, bench press, row, clean, and overhead press. No significant differences were found between KD and control groups, although both repetitions and total tonnage were higher in the KD group. The study of Wilson et al. [22] also evaluated the load volume (sets × repetitions × load); specifically, the loads increased between 2 and 5% during the last seven to nine weeks, but there were no significant differences between KD and control groups.

In Green’s study, which was carried out on CrossFit^®^ practitioners, no significant differences were found in volume load. Even when the total session time has been related to the volume load per muscle group, no differences have been found [23]. Recently, our research group evaluated parameters similar to those of previous studies, including the total number of repetitions and volume load in strength-trained participants [20]. We reported an increase in the number of repetitions (from 170.5 to 190.5) as well as in the volume load (from 325.6 to 350.4) from the first to the sixth week of applying the KD. Additionally, we incorporated other variables that may indicate performance during a session with KD application, such as the rate of perceived exertion (RPE) on a scale of 1 to 10, starting with values of 7.5 and ending with values of 7. Furthermore, using a linear encoder, we included two variables: velocity loss and effort index. While no differences were found in velocity loss, significant differences were observed in the effort index.

Therefore, based on our results, it seems that when it comes to promoting muscle hypertrophy in advanced participants, KD does not impair performance in a training session; however, it does not offer any added advantage either. The limited study and evaluation of 1-RM to assess these parameters requires more research that evaluates strength-trained participants’ performance in a session with the goal of hypertrophy under the prescription of KD.

## 5. Conclusions

We conclude that the KD does not impair BP or SQ 1-RM results in advanced participants, although it does not offer more favorable results either. In addition, the 1-RM test does not seem to be the best option to evaluate performance in a strength session with trained participants or athletes with a goal of muscle hypertrophy.

## Figures and Tables

**Table 1:** Characteristics of six randomized controlled clinical trials. FFM = fat free mass, LBM = lean body mass, FAT = fat adipose tissue, PRO = protein, CHO = carbohydrates, CKD = cyclical ketogenic diet, RDKD = reduction ketogenic diet, NKD = non-ketogenic diet, VLCKD = very low carbohydrate ketogenic diet, WD = Western diet, SQ = squat, BP = bench press, CMJ = countermovement jump, SJ = squat jump, PC = power clean, RM = repetition maximum, MVC = isometric contraction, N = Newton, RT = resistance training, M = men, W = women.

Reference	Sample	Duration	Nutritional Protocol	Country	Main Results	Measurement of Strength/Performance
Paoli et al., 2021 [34]	Male body-builders KD = 9; NKD = 10 Age KD = 26.2 ± 5.09; NKD = 31.67 ± 10.39 years. Weight KD = 86.39 ± 15.42; NKD = 89.04 ± 11.73. BMI KD = 26.97 ± 1.86; NKD = 26.66 ± 2.04 kg/m^2^	8 weeks	KD: CHO, 5%, less than 50 g/day PRO, 2.5 g∙kg^−1^·d^−1^ WD: CHO, 55% PRO, 2.5 g∙kg^−1^·d^−1^ The fats in both groups would be the calories until reaching 45 kcal/kg of muscle mass.	Italy	Maximal strength increased similarly in both groups.	1-RM Bench Press 1-RM Squat
Vargas-Molina et al., 2020 [21]	Resistance-trained women KD = 10; NKD = 11 Age KD = 26.8 ± 3.9; NKD = 28.3 ± 4.1 years. Weight KD = 61.9 ± 9.8; NKD = 62.6 ± 3.9 kg. BMI KD = 23.8 ± 3.6; NKD = 23.7 ± 2.2 kg/m^2^	8 weeks	40–45 kcal∙kg-FFM^−1^∙d^−1^ (KD: 1.7 g∙kg^−1^·d^−1^ PRO, 30–40 g∙kg∙d^−1^ CHO, remaining calories FAT; NKD: 1.7 g∙kg^−1^·d^−1^ PRO, 1 g∙kg^−1^·d^−1^ FAT, remaining calories CHO)	Spain	KD: No significant changes in BP. Significant changes in SQ and CMJ (5.6 kg/1.7 cm). NKD: Significant changes in BP, SQ, and CMJ (4.8 kg, 15.6 kg, 2.2 cm, respectively)	1-RM Bench Press 1-RM Squat CMJ
Kysel et al., 2020 [32]	25 recreational trained males in RT KD = 13; NKD = 12 Age CKD: 23.0 ± 5 and RD: 24.0 ± 4 years. Weight KD = 85.6 ± 13.4; NKD = 93 ± 17.5 kg. BMI KD = 26.1 ± 3.7; NKD = 26.9 ± 4.3 kg/m^2^	8 weeks	CKD: 5 days 30 g CHO, 1.6 g∙kg^−1^·d^−1^ PRO, rest FAT + 2 days 8–10 g∙kg^−1^·d^−1^ CHO (70% CHO, 15% PRO, 15% FAT) RD: 55% CHO, 30% FAT, 15% PRO. 500 Kcal deficit (CKL and RD)	Czech Republic	KD: No change in strength. NKD: Significant changes in Lat pull down (70.4/75.2) and Leg press (127.8/140).	1-RM Bench Press 1-RM Lat pull-down 1-RM Leg Press
Wilson et al., 2020 [22]	Resistance-trained males KD = 13; NKD = 12 Age KD: 23.0 ± 4.5 and NKD: 21.3 ± 3.7 years	10 weeks + 1 week = 11 weeks	KD = 5% CHO 20% PRO 75% FAT; NKD = 55% CHO, 20% PRO, 25% FAT	United States	KD: Bench Press, 252.7 to 275.38 Squat, 287.31 to 315.38 NKD: Bench Press, 248.8 to 265 Squat, 271.3 to 304.6 No differences between groups.	1-RM Bench press 1-RM Squat
LaFountain et al., 2019 [28]	Military health adults males/women KD = 15; NKD = 14 Age KD: 27.4 ± 6.8 and MD: 24.6 ± 9 years. Weight KD = 85.7 ± 7.8; NKD = 79.8 ± 5.5 kg. BMI KD = 27.9 ± 2.9; NKD = 24.9 ± 2.4 kg/m^2^	12 weeks	KD ≤ 50 g/day CHO 0.6–1.0 g∙kg^−1^·d^−1^ of LBM PRO Rest calories FAT Ad Libitum MD = maintained their habitual diet with a minimum consumption of ~40% CHO	United States	KD: SQ: Strength level was maintained (Pre: 117.6; Pos: 129.4 kg, *p* = 0.069). BP: Pre and Post 95.9 kg, *p* = 0.974) CMJ: Pre 34; Post 34.7 cm, *p* = 0.803) NKD: SQ: (Pre: 103, Post 122.4 Kg). BP: (Pre: 84.6, Post: 90.9 kg). CMJ: (Pre: 34.3, Post: 35.6 cm)	1-RM Bench press 1-RM Squat CMJ
Kephart et al., 2018 [33]	CrossFit-trained males/women (9 M, 3 W). KD = 7; NKD = 5. Age KD = 32 ± 3; NKD = 29 ± 3 years. Weight KD = 82.7 ± 8.2; NKD = 76.9 ± 5.5.	12 weeks	Not reported Ad Libitum	United States	No significant differences or changes between groups. SQ: *p* = 0.422 PC: *p* = 0.347	1-RM Back Squat 1-RM Power clean
